# 

**Published:** 1894-06

**Authors:** 


We have received the following entertaining numbers of The Physician's Leisure Library, Geo. S. Davis, Publisher, 1893:

"Antiseptic Therapeutics," in two volumes, by Dr. E. L. Trouessart, Paris, France. Translated by E. P. Hurd, M.D.

"Treatment of Typhoid Fever," by D. D. Stewart, M.D.

"The Modern Climatic Treatment of Invalids with Pulmonary Consumption in Southern California," by P. C. Remondino, M.D.

